# Indicators of patients with major depressive disorder in need of highly specialized care: A systematic review

**DOI:** 10.1371/journal.pone.0171659

**Published:** 2017-02-08

**Authors:** Frédérique C. W. van Krugten, Meriam Kaddouri, Maartje Goorden, Anton J. L. M. van Balkom, Claudi L. H. Bockting, Frenk P. M. L. Peeters, Leona Hakkaart-van Roijen, Decision Tool Unipolar Depression (DTUD) Consortium

**Affiliations:** 1 Institute of Health Policy & Management and Institute for Medical Technology Assessment, Erasmus University Rotterdam, Rotterdam, The Netherlands; 2 Department of Psychiatry and EMGO Institute for Health and Care Research, VU-University Medical Center and GGZ inGeest, Amsterdam, The Netherlands; 3 Department of Clinical Psychology, Utrecht University, Utrecht, The Netherlands; 4 Department of Psychiatry and Neuropsychology, Maastricht University, Maastricht, The Netherlands; Universita Cattolica del Sacro Cuore Sede di Roma, ITALY

## Abstract

**Objectives:**

Early identification of patients with major depressive disorder (MDD) that cannot be managed by secondary mental health services and who require highly specialized mental healthcare could enhance need-based patient stratification. This, in turn, may reduce the number of treatment steps needed to achieve and sustain an adequate treatment response. The development of a valid tool to identify patients with MDD in need of highly specialized care is hampered by the lack of a comprehensive understanding of indicators that distinguish patients with and without a need for highly specialized MDD care. The aim of this study, therefore, was to systematically review studies on indicators of patients with MDD likely in need of highly specialized care.

**Methods:**

A structured literature search was performed on the PubMed and PsycINFO databases following PRISMA guidelines. Two reviewers independently assessed study eligibility and determined the quality of the identified studies. Three reviewers independently executed data extraction by using a pre-piloted, standardized extraction form. The resulting indicators were grouped by topical similarity, creating a concise summary of the findings.

**Results:**

The systematic search of all databases yielded a total of 7,360 references, of which sixteen were eligible for inclusion. The sixteen papers yielded a total of 48 unique indicators. Overall, a more pronounced depression severity, a younger age of onset, a history of prior poor treatment response, psychiatric comorbidity, somatic comorbidity, childhood trauma, psychosocial impairment, older age, and a socioeconomically disadvantaged status were found to be associated with proxies of need for highly specialized MDD care.

**Conclusions:**

Several indicators are associated with the need for highly specialized MDD care. These indicators provide easily measurable factors that may serve as a starting point for the development of a valid tool to identify patients with MDD in need of highly specialized care.

## Introduction

Major depressive disorder (MDD) is one of the most prevalent psychiatric disorders [1,2] and is projected to be the leading cause of disease burden in high-income countries by 2030 [3]. MDD presents many treatment challenges, not the least of which is the subset of patients with depression that is refractory to secondary mental health services. Often, these patients receive inadequate, too low-intensity treatment in secondary mental health services [4–7], which is associated with a longer treatment course [8], an increased risk of suicide [9–11] and substantial societal costs [12,13].

Early identification of patients with MDD who cannot be managed by secondary services and require highly specialized care could enhance need-based patient stratification. This, in turn, may reduce the number of treatment steps needed to achieve and sustain an adequate treatment response, and may subsequently benefit the quality of life of patients. To date, validated tools to facilitate need-based patient stratification are rarely used in psychiatric practice. This is in marked contrast to other areas of medicine such as oncology [14–18], in which patient stratification on the basis of clinical presentation plays an important role in treatment planning from the time of diagnosis.

The development of a validated tool to identify patients with MDD in need of highly specialized care during the diagnostic phase after referral is hampered by the lack of a comprehensive understanding of the indicators that distinguish patients with and without a need for highly specialized MDD care. There are several reviews available which summarize the studies on factors associated with a recurrent or persistent clinical course [19–22] for which more intensive treatment is indicated [23]. However, to date none have focused on the factors associated with a broad range of unfavourable clinical outcomes, thereby preventing the construction of an overall picture of the indicators of patients with MDD in need of highly specialized care. Therefore, the aim of this study is to systematically review studies on indicators of patients with MDD likely in need of highly specialized care.

## Methods

### Definition of terms

For the purpose of this study, primary mental healthcare is defined as the care provided to people with mental health problems within the primary care setting. Secondary mental healthcare is delivered primarily through community mental health services and psychiatric services in general hospitals, and refers to the more specialized support provided to patients with mental health needs that cannot be supported by primary care services [24]. Highly specialized mental healthcare, also commonly referred to as tertiary mental healthcare, is defined as specialized intervention delivered by highly-trained staff with specific expertise in a given field to individuals with problems that cannot be treated with sufficient result by either primary or secondary mental health services [25,26]. Finally, the term indicators is used to refer to clinical characteristics and risk factors that may aid clinicians in the identification of the subgroup of patients with MDD likely in need of highly specialized care.

### Expert input and proxy indicators of need for highly specialized MDD care

Prior to performing the structured literature search, the Decision Tool Unipolar Depression Consortium was formed comprising thirteen leading MDD experts from six independent psychiatric specialized and highly specialized mental healthcare clinics across the Netherlands. The consortium of experts assisted with refining the research question and provided guidance for the conduct of the literature search. In the absence of studies directly examining clinical and sociodemographic factors associated with a need for highly specialized MDD care, proxy indicators had to be identified. In a digital survey, consortium members and a number of other qualified domain experts were asked to define terms by which (the clinical course of) patients with MDD in need of highly specialized care can be described (hereafter named proxy indicators of need for highly specialized MDD care). All domain experts were required to have specialist expertise regarding the research question, as evidenced by the fact that they were either active as a clinician or researcher in the field of depression. Ultimately, via existing national depression networks, 134 experts were approached, 67 of whom participated in the study. After an analysis of the concepts submitted by the experts, four high-frequency proxy indicators of need for highly specialized MDD care were selected. The proxy indicators of need for highly specialized MDD care that were selected for the purpose of this review include: "Treatment-Resistant", "Chronic", "Recurrent", and "Persistence of Severity".

### Eligibility criteria

Studies were selected for review if they met the following inclusion criteria:

1. Published in English or Dutch, related to humans and full-text was available
2. Published between January 2000 and January 2015
3. The study design was either a randomized controlled trial, case-control study, cross-sectional study or cohort study
4. The study was an investigation of (a group of) adult psychiatric patients (aged 18 and over) with MDD as their primary diagnosis according to the Diagnostic and Statistical Manual of Mental Disorders (DSM)-III [27], DSM-III-R [28], DSM-IV [29], DSM-IV-TR [30], DSM-5 [31], International Classification of Diseases (ICD)-9 [32], ICD-10 [33] or Research Diagnostic Criteria (RDC) [34]
5. The main outcome variable used was one of the four following proxy indicators of need for highly specialized MDD care: "Treatment-Resistant", "Chronic", "Recurrent", and "Persistence of Severity"
6. One of the aims of the study was to identify clinical and/or sociodemographic factors that discriminate MDD patients with a proxy of need for highly specialized care (i.e. "cases") from those without a proxy indicator of need for highly specialized care (i.e. "non-cases")

This study is restricted to indicators of patients with MDD in need of highly specialized care, which can be assessed during the diagnostic phase after referral. Hence, no papers that solely reported on physiological, neurobiological, or genetic factors were eligible for inclusion. Furthermore, since the aim was to identify indicators of patients with an unfavourable treatment course treated in secondary mental health services who may benefit from highly specialized care, we excluded studies focusing exclusively on participants from primary care populations or the general population.

### Data sources and search strategy

To identify studies reporting indicators of patients with MDD in need of highly specialized care, a structured literature search was performed on the PubMed (National Library of Medicine) and PsycINFO (Ovid) databases following PRISMA guidelines [17]. The search for published primary articles was conducted on January 15, 2015 and was restricted to articles written in English or Dutch, published between January 1, 2000 and January 15, 2015, related to humans and of which the full text was available. Search terms were chosen based on the proxy indicators of need for highly specialized MDD care as defined by domain experts. The Medical Subject Headings (MeSH) of relevance to this review included the following search terms: "depressive disorder", "depression" and "depressive disorder, treatment-resistant". In addition, keywords were searched within the title or full-text. Keywords included: "chronic", "chronic depression", "chronicity", "recurrent", "recurrent depression", "recurring", "severe", "severe depression" and "severity". A complete list of search strategies can be found in the S1 Tables. We did not register a systematic review protocol.

### Study selection

Prior to examining all articles identified through the primary search, two reviewers independently screened a random sample of 66 titles and abstracts whilst blinded to authors and journal titles, and reached strong agreement (Cohen's κ = 0.85) using an Excel workbook designed for this purpose [35]. They then independently screened all records whilst still blinded to authors and journal titles. Full papers were retrieved for all references that had been judged as potentially eligible and were examined independently by two researchers. Disagreements were resolved by discussion or through third party adjudication.

### Data abstraction

Three reviewers independently executed data extraction by using an Excel-based, pre-piloted, standardized extraction form. Disagreements were resolved by discussion between the three reviewers. The following characteristics of the studies were coded: (1) general study characteristics (author, year of publication, country); (2) characteristics of the study population (sample size, age of inclusion, mean age, number of MDD patients with and without a proxy of need for highly specialized care); (3) design of the study (case-control, cross-sectional or longitudinal); (4) depression measure and proxy of need for highly specialized MDD care (e.g. treatment-resistant, recurrence); (5) clinical and/or sociodemographic factors on which MDD patients with and without a proxy of need for highly specialized care significantly differed. If results from a multivariable regression analysis were available, then those findings were included rather than bivariate results. If results from several regression models were presented, only results from the model with the largest number of predictors were used. The purpose of this review was to identify, rather than quantify, the factors associated with proxies of need for highly specialized care. Thus, a meta-analysis was not performed and data were synthesized in a narrative review. In a consensus-building process, the experts categorized the abstracted indicators by topical similarity, creating a concise summary of the findings. The resulting categories were identified as the overarching indicators of patients with MDD in need of highly specialized care, provided that the direction of association between the indicators grouped within the category and proxies of need for highly specialized care was consistent. Within the categories grouping indicators with opposite directions (e.g. low and high educational level), subcategories of indicators with a consistent direction of association were identified as the indicators of patients with MDD in need of highly specialized care.

### Quality assessment

Two reviewers independently evaluated the methodological quality of the included studies using the 14-item National Heart, Blood and Lung Institute (NHBLI) Quality Assessment Tool for Observational Cohort and Cross-Sectional Studies [36] or the 12-item NHBLI Quality Assessment Tool for Case-Control Studies [37]. Each of the items was scored as "yes", "no", "not reported" or "not applicable" on the basis of the information provided in the article. Disagreements were resolved by discussion or through third party adjudication. A quality score, expressed as a percentage of the maximum possible score, was calculated for each study.

## Results

### Study selection

**Fig 1:**
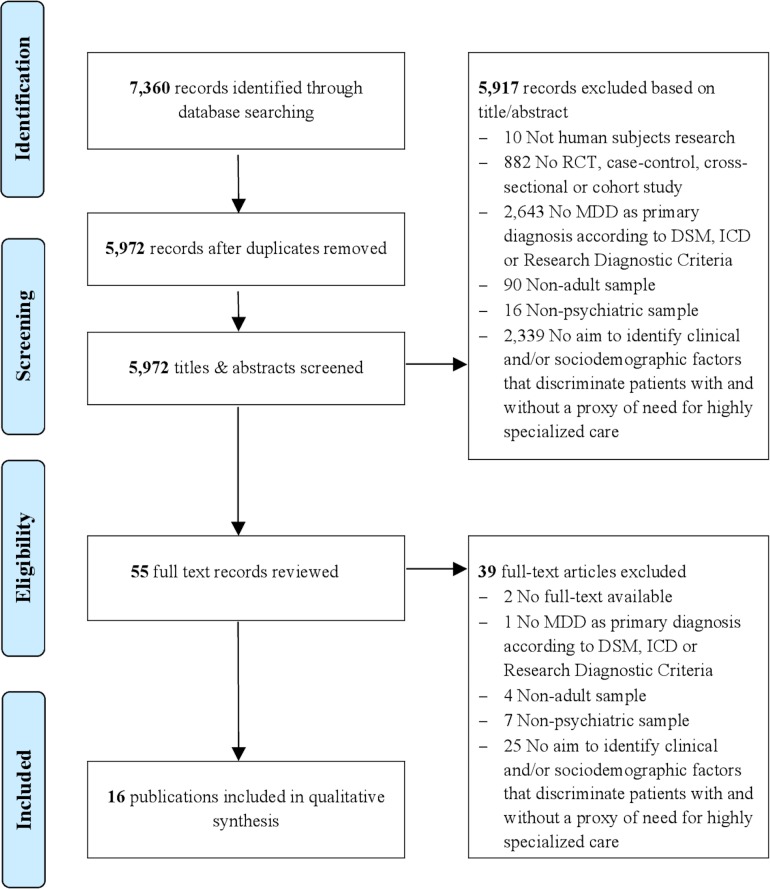
Flow chart of study selection process. RCT, Randomized controlled trial; MDD, major depressive disorder; DSM, Diagnostic and Statistical Manual of Mental Disorders; ICD, International Classification of Diseases.

The systematic search of all databases yielded a total of 7,360 references. Duplicates were checked and excluded (n = 1,388). Title and abstract screening resulted in the exclusion of a further 5,917 papers. Main reasons for exclusion were that papers: had a design other than a randomized controlled trial, case-control study, cross-sectional study or cohort study; had an initial population or control group other than subjects with MDD as their primary diagnosis; had an aim other than the identification of clinical and/or sociodemographic factors that discriminate patients on the basis of a proxy of need for highly specialized care. Full texts of the remaining 55 papers were obtained for detailed review. Thirty-nine papers were excluded following full text screening. Sixteen papers fulfilled the eligibility criteria and were incorporated into this review. Details of the study selection process are provided in Fig 1.

### Study characteristics

The general characteristics of the included papers are presented in Table 1. Six papers focused on treatment-resistant depression [38–43], four on chronic depression [44–47], five on recurrence in depression [48–52], and one on persistence of severity [53]. The vast majority of included papers utilized cross-sectional data. Most of the included studies were conducted in the United States (n = 5) and Europe (n = 8) with the remainder in Asia (n = 3).

**Table 1:** General characteristics of the included studies.

Outcome examined and study	Population	Sample size (C/NC)	Mean age C/NC (SD)	Diagnostic criteria	Study design (follow-up length)	Country	NHBLI Quality Score (%)
**Treatment-resistance**							
Kaplan et al. 2000 [38]	Outpatients from a university clinic	40 (20/20)	47 (*not given*) / 45 (*not given*)	DSM-IV and ICD-10	Nested case-control	USA	50
Souery et al. 2007 [39]	Outpatients and inpatients from specialist referral centres	702 (356/346)	50.5 (14.1) / 51.5 (14.6)	DSM-IV	Cross-sectional	European countries	57
Amital et al. 2008 [40]	Outpatients from community psychiatric clinics	107 (42/65)	54.7 (16.3) / 49.6 (16.2)	ICD-10	Cross-sectional	Israel	43
Dudek et al. 2010 [41]	Outpatients from psychiatric clinics	1,051 (570/481)	47 (11) / 46 (11)	DSM-IV-TR	Cross-sectional	Poland	36
Takahashi et al. 2013 [42]	Outpatients from university clinics	62 (35/27)	38.74 (9.42) / 39.07 (9.19)	DSM-IV	Cross-sectional	Japan	36
Takahashi et al. 2013 [43]	Outpatients from university clinics	66 (35/31)	35.94 (8.93) / 38.00 (8.42)	DSM-IV	Cross-sectional	Japan	36
**Chronicity**							
Riso et al. 2003 [44]	Outpatients from a university mood disorders unit	69 (42/27)	39.3 (10.3) / 39.1 (10.3)	DSM-IV	Cross-sectional	USA	43
Gilmer et al. 2005 [45]	Outpatients from primary or psychiatric care sites	1,380 (293/1,087)	41.9 (13.5) / 39.7 (13.0)	DSM-IV	Cross-sectional	USA	43
Wiersma et al. 2009 [46]	Subjects from the community, primary care settings, and specialized mental healthcare facilities	1,204 (395/809)	42.4 (11.8) / 39.7 (12.3)	DSM-IV	Cross-sectional	NL	50
Wiersma et al. 2011 [47]	Subjects from the community, primary care settings, and specialized mental healthcare facilities	1,002 (312/690)	40.5 (12.2) / 43.2 (11.8)	DSM-IV	Cross-sectional	NL	50
**Recurrence**							
Melartin et al. 2004 [48]	Secondary-level care psychiatric outpatients and inpatients	198 (76/122)	41.0 (11.1)^a^	DSM-IV	Longitudinal (18 months)	Finland	86
Solomon et al. 2004 [49]	Outpatients and inpatients from academic medical centers	290 (143/147)	39 (15)^a^	RDC	Longitudinal (15 years)	USA	71
Bos et al. 2005 [50]	Female outpatients	50 (30/20)	*Not given*	DSM-IV	Cross-sectional	NL	43
Hollon et al. 2006 [51]	Outpatients from primary and psychiatric care sites	1,426 (1,061/365)	41.2 (13.2) / 38.9 (13.4	DSM-IV	Cross-sectional	USA	50
Gerrits et al. 2014 [52]	Subjects from the community, primary care settings, and specialized mental healthcare facilities	*Not given* (292/*not given*)	43.4 (12.8)^b^	DSM-IV	Longitudinal (4 years)	NL	71
**Persistence of severity**							
Lamers et al. 2011 [53]	Subjects from the community, primary care settings, and specialized mental healthcare facilities	789 (19%/81%)	41.8 (12.0)^a^	DSM-IV	Longitudinal (1 year)	NL	64

C/NC, Cases/Non-Cases; SD, standard deviation; NHBLI, National Heart, Blood and Lung Institute; DSM, Diagnostic and Statistical Manual of Mental Disorders; ICD, International Classification of Diseases; RDC, Research Diagnostic Criteria; USA, United States of America; NL, The Netherlands.

^a^ For the overall sample.

^b^ For the overall sample (n = 1122), including anxiety patients.

### Methodological quality of the included studies

The overall quality scores are presented in Table 1; quality scores for the separate NHBLI criteria are shown in the S2 Tables. The overall quality scores ranged from 36% ([41–43]) to 86% ([48]). The main issues with included articles were the lack of sample size justification and the lack of repeated exposure assessment. The research question and study population were clearly defined in the majority of the included studies.

### Indicators of need of highly specialized care

Table 2 lists the indicators of patients with a depression in need of highly specialized care. The sixteen papers yielded a total of 48 unique clinical and sociodemographic factors on which MDD patients with and without a proxy of need for highly specialized care significantly differed. In general, the abstracted clinical and sociodemographic factors could be grouped into the following seven categories: depression severity, onset and (treatment) course, comorbid psychopathology, somatic comorbidity, childhood trauma, psychosocial functioning, and sociodemographics. Each of the abstracted indicators will be discussed in the following sections.

**Table 2:** Indicators of patients with a depression in need of highly specialized care.

Indicator
**Depression severity**
Greater (baseline [53]) depressive symptom severity [46,48]
Current suicidal risk [39]
Higher rates of melancholic features [39]
Higher levels of rumination [47]
**Onset and (treatment) course**
Younger age of onset [41,51]
Longer time since first onset [51]
History of prior suicide attempts [45]
Shorter current episode [51]
Less likely to meet criteria for chronic depression [51]
More than three previous depressive episodes [41]
Fewer prior episodes of depression [45]
Lack of remission or partial remission after the previous depressive episode [41]
Nonresponse to first antidepressant treatment lifetime [39]
**Comorbid psychopathology**
A higher number of comorbid psychiatric disorders [48]
Comorbid (generalized [45]) anxiety disorder [38,39,46]
Higher levels of chronic PTSD [38]
More symptoms of bipolarity [41]
Higher scores on the MMPI-2 subscales [38]
Lower levels of extraversion on the NEO-FFI [42,47]
Lower levels of reward dependence on the TCI-125 [43]
Lower levels of self-directedness on the TCI-125 [43]
Higher levels of harm avoidance on the TCI-125 [43]
Higher levels of impaired autonomy on the YSQ [44]
Higher levels of disconnection and rejection on the YSQ [44]
Higher levels of overvigilance on the YSQ [44]
Higher levels of external locus of control on the SMS [47]
Higher levels of neuroticism on the NEO-FFI [53]
**Somatic comorbidity**
Greater general medical comorbidity [45]
Worse physical health function [45]
Lower physical quality of life [45]
Severe neck, chest and abdominal pain [52]
A higher number of pain locations [52]
Higher pain severity of pain [52]
**Childhood trauma**
Higher prevalence of childhood trauma [46]
Greater levels of childhood emotional abuse [38]
Higher levels of trauma sequelae [38]
**Psychosocial functioning**
Worse work function and social adjustment [45]
Impaired psychosocial functioning [49]
Lower quality of life [45]
**Sociodemographics**
Older age [45,50,51]
Less education [45]
Higher educational level [50]
Lower monthly household income [45]
No private insurance [45]
Unemployment [45]
Prior job loss [40]
A greater likelihood of being Black as opposed to white or other [45]
A greater likelihood of being Hispanic as opposed to non-Hispanic [45]

MMPI-2, Minnesota Multiphasic Personality Inventory-2; NEO-FFI, NEO Five-Factor Inventory; TCI-125, Cloninger's 125-question Temperament and Character Inventory; YSQ, Young Schema Questionnaire; SMS, Self-Mastery Scale.

#### Depression severity

A more pronounced depression severity, whether operationalized by the number or type of symptoms, has consistently been shown to be associated with proxies of need for highly specialized MDD care. Three studies reported a correlation between higher levels of (baseline [53]) depressive symptomatology and proxies of need for highly specialized MDD care [46,48]. In addition, the presence of certain symptoms such as current suicidal risk [39], an increased likelihood of melancholic features [39], and higher levels of rumination [47] were found to be associated with proxies of need for highly specialized MDD care.

#### Onset and (treatment) course

Patients with a proxy of need for highly specialized care were found to have an earlier age of onset of the first major depressive episode [41,51], and subsequently reported a longer time since first onset of MDD [51]. In addition, factors denoting a history of poor treatment response such as nonresponse to first antidepressant received [39], and lack of remission or partial remission after the previous depressive episode [41] were found to be associated with proxies of need for highly specialized care. Inconsistent results were found for the number of prior episodes of depression [41,45].

#### Comorbid psychopathology

There have been several studies that examined the association between comorbid psychopathology and proxies of need for highly specialized care. Melartin et al. [48] found that the presence of a higher number of comorbid psychiatric disorders in general increases the risk of recurrence. In addition, the following specific comorbid psychiatric disorders were found to be associated with proxies of need for highly specialized care: chronic PTSD [38], (generalized [45]) anxiety disorder [38,39,46], and more symptoms of bipolarity [41]. Furthermore, relations between psychopathological dimensional personality traits and proxies of need for highly specialized care have been found repeatedly. In two closely related articles, Takahashi et al. [42,43] reported that high scores for harm avoidance, low scores for reward dependence, low scores for self-directedness, and low scores for extraversion are personality dimensions in patients with treatment-resistant depression. In addition, Kaplan and Klinetob [38] reported that patients with treatment-resistant depression had clinically significant elevations on the Minnesota Multiphasic Personality Inventory-2 (MMPI-2, [54]) subscales hypochondriasis, depression, hysteria, psychopathic deviate, paranoia, psychasthenia, schizophrenia and social introversion. Further, higher levels of impaired autonomy [44], higher levels of disconnection and rejection [44], higher levels of overvigilance [44], higher levels of external locus of control [47], and higher levels of neuroticism [53] have been linked to proxy indicators of need for highly specialized care.

#### Somatic comorbidity

Increased general medical comorbidity [45], severe neck, chest and abdominal pain [52], a higher number of pain locations [52] and higher pain severity of pain [52] were found to be associated with proxies of need for highly specialized care. Subsequently, lower levels of physical health function [45] and a lower physical quality of life [45] have been linked to proxy indicators of need for highly specialized care.

#### Childhood trauma

Two studies [38,46] examined the relationship between childhood trauma and a proxy indicator of need for highly specialized care. In a sample of 1,230 individuals, Wiersma et al. [46] examined the relationship between retrospective reports of childhood life events and childhood trauma and the risk of chronicity of MDD in adulthood. They found that a reported history of multiple childhood traumas, such as emotional neglect, psychological abuse, physical abuse, and sexual abuse, was associated with chronicity of depression. Kaplan and Klinetob [38] similarly found that patients with treatment-resistant depression reported more emotional abuse and experienced current-day trauma sequelae when compared to treatment responders.

#### Psychosocial functioning

Two of the included studies [45,49] reported that patients with a proxy of need for highly specialized care were more likely to exhibit impaired functioning in areas such as work, relationships and leisure. Moreover, a poorer quality of life, as operationalized by the Quality of Life Enjoyment and Satisfaction Questionnaire (QLESQ, [55]), was found to be associated with a proxy of need for highly specialized care [45].

#### Sociodemographics

Many studies examined the associations between sociodemographic factors and proxies of need for highly specialized care. Three papers [45,50,51] reported an association between older age and a proxy of need for highly specialized care. In addition, individuals with a proxy of need for highly specialized care were found to be socioeconomically disadvantaged when compared to individuals without a proxy of need for highly specialized care [45]. Contrasting findings were found for educational level. One study [45] reported that patients with a lower level of education exhibited greater chronicity than patients with a higher level of education. By contrast, the study by Bos et al. [50] found the reverse: patients with a history of recurrent depression were more highly educated compared to individuals with a single episode.

## Discussion

The aim of this systematic review was to identify indicators of patients with MDD in need of highly specialized care. Overall, a more pronounced depression severity, a younger age of onset, a history of prior poor treatment response, psychiatric comorbidity, somatic comorbidity, childhood trauma, psychosocial impairment, older age, and a socioeconomically disadvantaged status were found to be associated with proxies of need for highly specialized MDD care.

To our knowledge, this is the first systematic literature search that comprehensively covers the factors associated with a broad range of unfavourable clinical outcomes in patients with MDD for which more intensive treatment is indicated [23]. To date, reviews solely summarized factors associated with one of the proxy indicators of need for highly specialized care [19–22], thereby preventing the construction of an overall picture of the indicators of patients with MDD in need of highly specialized care. Our systematic and comprehensive review allows the delineation of this subgroup of patients, makes them identifiable, and thus adds to the process of further professionalizing and improving quality in the mental healthcare sector.

This study has several limitations. First, this study does not shed light on the efficacy of highly specialized care in meeting patients' treatment needs. Although highly specialized care has been demonstrated to improve clinical outcomes in patients with complex and severe conditions in other areas of medicine [56], the net benefit of highly specialized care in patients with MDD has not yet been studied. However, the evaluation of the impact of highly specialized care on patient outcomes in this population is of utmost importance and should therefore be addressed in future studies. Second, the focus of this systematic review was to identify indicators that could be easily assessed in routine clinical practice, specifically during the diagnostic phase after referral. This resulted in the exclusion of papers solely reporting on physiological, neurobiological, and genetic patient factors, making it possible that other indicators with strong evidence for a need for highly specialized MDD care have been missed. Third, due to considerable heterogeneity of populations, sample sizes, range of predictors, outcomes and statistical analyses no quantitative synthesis of the results in a meta-analysis could be performed. Fourth, since the aim of this study was to assess the current state of research on indicators of patients with an unfavourable treatment course treated in secondary mental health services who may benefit from highly specialized care, we also included studies with a heterogeneous mixture of patients from the community and from primary and psychiatric care sites, as they contained a subgroup of psychiatric patients. This may have influenced the results, as recent studies suggest that the determinants and nature of the long-term course of depression of subjects from the community and primary sites differentiates from that of patients from psychiatric care sites [57–59]. However, an additional qualitative synthesis of the results in the subset of studies exclusively reporting on patients with MDD treated in psychiatric care sites did not alter the results, suggesting that the associations between indicators of need for highly specialized care are similar for psychiatric and non-psychiatric patients.

On the basis of this review, we posit the primary importance of the following nine indicators of patients with MDD in need of highly specialized care: a more pronounced depression severity, a younger age of onset, a history of prior poor treatment response, psychiatric comorbidity, somatic comorbidity, childhood trauma, psychosocial impairment, older age, and a socioeconomically disadvantaged status. It should be noted, however, that these indicators alone are not likely to justify referral to highly specialized mental healthcare programs. Rather, in combination with one another they may provide healthcare practitioners with a guideline for determining the need for highly specialized care. Future research should explore how the identified set of indicators can facilitate the early identification of patients with MDD in need of highly specialized care. In addition, we believe that advances in the development of a valid tool to identify patients with MDD in need of highly specialized care during the diagnostic phase after referral will need to be based on more refined, better operationalized indicators. Furthermore, while the identified indicators have received the strongest support in the literature, this may partly be due to the fact that they have received more research attention. It is therefore possible that other characteristics of patients in need of highly specialized care may theoretically be very important, but have not yet been sufficiently researched. Hence, in accordance with evidence-based medicine [60], this set of characteristics should be critically appraised, refined, and, if necessary, complemented by clinical expertise before applying review findings to clinical practice. The identified set of indicators may therefore serve as a starting point for the development of a valid tool to identify patients with MDD in need of highly specialized care during the diagnostic phase after referral. This may ultimately facilitate early detection and assist clinicians in selecting the most appropriate treatment option in a given clinical situation, thereby reducing the functional impact and socioeconomic burden of MDD.

## Supporting information

S1 Tables Search strategy. (DOCX)

S2 Tables Quality assessment. (DOCX)

S1 Table PRISMA checklist. (DOCX)
