# Supplementary material for: Indicators of patients with major depressive disorder in need of highly specialized care: A systematic review
Source: PLoS One. 2017 Feb 8;12(2):e0171659. doi: 10.1371/journal.pone.0171659 (PMC5298252; doi:10.1371/journal.pone.0171659)
Supplement: S1 Tables — (DOCX) [file pone.0171659.s001.docx]

**S1 Tables. Search strategy.**

**Pubmed (NLM)**

| **#** | **Searches** |
| --- | --- |
| 1 | ("Depressive Disorder"[Mesh major topic] OR "Depression"[Mesh major topic]) AND ("Depressive Disorder, Treatment-Resistant"[Mesh major topic] OR chronic[ti] OR “chronic depression” OR chronicity[ti] OR complex[ti] OR “complex depression” OR complexity[ti] OR complicated[ti] OR recurrent[ti] OR “recurrent depression” OR recurring[ti] OR severe[ti] OR “severe depression” OR severity[ti]) |
| 2 | limit 1 to full text |
| 3 | limit 2 to human |
| 4 | limit 3 to english or Dutch language |
| 5 | limit 101 to yr="2000 -Current" |

**PsycINFO (Ovid)**

| **#** | **Searches** |
| --- | --- |
| 1 | "depressive disorder".ti,ab. |
| 2 | "disorder, depressive".ti,ab. |
| 3 | "disorders, depressive".ti,ab. |
| 4 | "neurosis, depressive".ti,ab. |
| 5 | "depressive neuroses".ti,ab. |
| 6 | "depressive neurosis".ti,ab. |
| 7 | "neuroses, depressive".ti,ab. |
| 8 | "depression, endogenous".ti,ab. |
| 9 | "endogenous depression".ti,ab. |
| 10 | "endogenous depressions".ti,ab. |
| 11 | "depressive syndrome".ti,ab. |
| 12 | "depressive syndromes".ti,ab. |
| 13 | "syndrome, depressive".ti,ab. |
| 14 | "syndromes, depressive".ti,ab. |
| 15 | "depression, neurotic".ti,ab. |
| 16 | "depressions, neurotic".ti,ab. |
| 17 | "neurotic depression".ti,ab. |
| 18 | "neurotic depressions".ti,ab. |
| 19 | "melancholia".ti,ab. |
| 20 | "melancholias".ti,ab. |
| 21 | "unipolar depression".ti,ab. |
| 22 | "depression, unipolar".ti,ab. |
| 23 | "depressions, unipolar".ti,ab. |
| 24 | "unipolar depressions".ti,ab. |
| 25 | 1 or 2 or 3 or 4 or 5 or 6 or 7 or 8 or 9 or 10 or 11 or 12 or 13 or 14 or 15 or 16 or 17 or 18 or 19 or 20 or 21 or 22 or 23 or 24 |
| 26 | depression.ti,ab. |
| 27 | depressions.ti,ab. |
| 28 | "depressive symptoms".ti,ab. |
| 29 | "depressive symptom".ti,ab. |
| 30 | "symptoms, depressive".ti,ab. |
| 31 | "emotional depression".ti,ab. |
| 32 | "depression, emotional".ti,ab. |
| 33 | "emotional depressions".ti,ab. |
| 34 | 26 or 27 or 28 or 29 or 30 or 31 or 32 or 33 |
| 35 | "depressive disorder, treatment-resistant".ti,ab. |
| 36 | "depressive disorders, treatment resistant".ti,ab. |
| 37 | "treatment-resistant depressive disorders".ti,ab. |
| 38 | "treatment-resistant depressive disorder".ti,ab. |
| 39 | "therapy-resistant depression".ti,ab. |
| 40 | "therapy resistant depression".ti,ab. |
| 41 | "therapy-resistant depressions".ti,ab. |
| 42 | "treatment resistant depression".ti,ab. |
| 43 | "depression, treatment resistant".ti,ab. |
| 44 | "resistant depression, treatment".ti,ab. |
| 45 | "treatment resistant depressions".ti,ab. |
| 46 | "refractory depression".ti,ab. |
| 47 | "depression, refractory".ti,ab. |
| 48 | chronic.ti. |
| 49 | "chronic depression".mp. [mp=title, abstract, heading word, table of contents, key concepts, original title, tests & measures] |
| 50 | chronicity.ti. |
| 51 | recurrent.ti. |
| 52 | "recurrent depression".mp. [mp=title, abstract, heading word, table of contents, key concepts, original title, tests & measures] |
| 53 | recurring.ti. |
| 54 | severe.ti. |
| 55 | "severe depression".mp. [mp=title, abstract, heading word, table of contents, key concepts, original title, tests & measures] |
| 56 | severity.ti. |
| 57 | 25 or 34 |
| 58 | 35 or 36 or 37 or 38 or 39 or 40 or 41 or 42 or 43 or 44 or 45 or 46 or 47 or 48 or 49 or 50 or 51 or 52 or 53 or 54 or 55 or 56 or 57 |
| 59 | 57 and 58 |
| 60 | limit 98 to full text |
| 61 | limit 99 to human |
| 62 | limit 100 to english or dutch language |
| 63 | limit 101 to yr="2000 -Current" |
